# Pre-statistical harmonization of behavrioal instruments across eight surveys and trials

**DOI:** 10.1186/s12874-021-01431-6

**Published:** 2021-10-25

**Authors:** Diefei Chen, Eric Jutkowitz, Skylar L. Iosepovici, John C. Lin, Alden L. Gross

**Affiliations:** 1 Department of Epidemiology, Johns Hopkins Bloomberg School of Public Health, Baltimore, MD, United States, 2024 E. Monument Street, Baltimore, MD 21205 USA; 2 Health Services, Policy & Practice, Brown School of Public Health, Providence, RI USA; 3 Center of Innovation in Long Term Services and Supports, Providence VA Medical Center, Providence, RI USA; 4 Johns Hopkins University Center on Aging and Health, Baltimore, MD USA

**Keywords:** ADRD, Dementia, Behavioral symptom management, Epidemiology, Pre-statistical harmonization

## Abstract

**Background:**

Data harmonization is a powerful method to equilibrate items in measures that evaluate the same underlying construct. There are multiple measures to evaluate dementia related behavioral symptoms. Pre-statistical harmonization of behavioral instruments in dementia research is the first step to develop a statistical crosswalk between measures. Studies that conduct pre-statistical harmonization of behavioral instruments rarely document their methods in a structured, reproducible manner. This is a crucial step which entails careful review, documentation and scrutiny of source data to ensure sufficient comparability between items prior to data pooling. Here, we document the pre-statistical harmonization of items measuring behavioral and psychological symptoms among people with dementia. We provide a box of recommended procedure for future studies.

**Methods:**

We identified behavioral instruments that are used in clinical practice, a national survey, and randomized trials of dementia care interventions. We rigorously reviewed question content and scoring procedures to establish sufficient comparability across items as well as item quality prior to data pooling. Additionally, we standardized coding to Stata-readable format, which allowed us to automate approaches to identify potential cross-study differences in items and low-quality items. To ensure reasonable model fit for statistical co-calibration, we estimated two-parameter logistic Item Response Theory models within each of the eight studies.

**Results:**

We identified 59 items from 11 behavioral instruments across the eight datasets. We found considerable cross-study heterogeneity in administration and coding procedures for items that measure the same attribute. Discrepancies existed in terms of directionality and quantification of behavioral symptoms for even seemingly comparable items. We resolved item response heterogeneity, missingness and skewness, conditional dependency prior to estimation of item response theory models for statistical co-calibration. We used several rigorous data transformation procedures to address these issues, including re-coding and truncation.

**Conclusions:**

This study highlights the importance of each aspect involved in the pre-statistical harmonization process of behavioral instruments. We provide guidelines and recommendations for how future research may detect and account for similar issues in pooling behavioral and related instruments.

**Supplementary Information:**

The online version contains supplementary material available at 10.1186/s12874-021-01431-6.

## Background

Often there are multiple instruments that evaluate the same underlying construct. Data harmonization is a powerful method that combines data obtained from different items that represent the same underlying construct. For example, in dementia research, behavioral symptoms are collected using different measures such as Neuropsychiatric Inventory-Questionnaire (NPI) and Problem Behavior Checklist. Such instruments can be combined by anchoring on the shared behavioral symptomology. Integrating data from different study populations encourages collaborations, increases sample size and statistical power, improves generalizability, facilitates subgroup analyses and investigation of rare phenotypes [21, 23, 24], ensures reliability of published study results [9], and optimzies existing data and research infrastructures [17]. Data harmonization has been used to advance research in genome-wide association studies [8, 26], neuroimaging [42], and dementia care [20, 25].

There are several approaches to data harmonization. For example, Item Response Theory (IRT) consists of modeling a latent variable based on different sets of items that represent the same underlying construct. Items can be measured within studies by different instruments or across studies [21, 23, 24]. Other commonly used statistical methods include standardization and missing data by design with multiple imputation [18]. Regardless of the statistical harmonization method of choice, pooling of data is complex and requires careful scrutiny of source datasets and items [2]. Most methods require data to have some common items to be used for linking purposes---this necessitates undertaking the qualitative process of pre-statistical harmonization [17]. However, this crucial step for optimizing existing research resources and infrastructures is rarely described in research.

Pre-statistical harmonization is the series of procedures undertaken before data pooling. The goal of pre-statistical harmonization is to identify items that are likely comparable across studies [17]. Pre-statistical harmonization involves selection of participant studies (e.g., careful review of study design, methods and study population), and identification of items to be harmonized [11]. It is crucial to identify items that are measured using comparable instruments. Candidate items for linking can be those measuring a comparable underlying construct, and can be harmonized using a simple transformation algorithm via IRT or other approaches. Several studies have described pre-statistical harmonization from disease areas including substance use [40] and cognitive impairment [6, 17].

In this study, we document the pre-statistical harmonization of dementia behavioral symptom measures captured in National Institutes of Aging funded Alzheimer’s Disease Research Centers clinical evaluations, a National Institutes of Aging national survey of cognitive health, and six National Institutes of Aging funded randomized trials of nondrug dementia care interventions that include assessments of dementia related behaviors. This study is the first step in a larger initiative to develop a statistical crosswalk between the different measures of dementia related behaviors administered in clinical practice, national surveys, and randomized trials of dementia care interventions. A major challenge in harmonization of behavioral data is the remarkable variability in questions across instruments and how they are asked. Studies vary in terms of response options, directionality in coding responses (e.g. 0 = No, 1 = Yes vs. 0 = Yes, 1 = No), quantification of behavioral manifestations, and other factors. To ensure the quality and reproducibility of data pooling, careful scrutiny of items to be harmonized is a crucial step to account for these differences. However, this phase of data harmonization is rarely discussed in published research.

In this paper, we aim to describe procedures we undertook during the pre-statistical harmonization process of measures of dementia related behaviors. We do so for the sake of reproducibility and to provide a guide for future studies requiring the consolidation of multiple data sources to evaluate behavioral symptoms of dementia [38, 39]. Specifically, we describe approaches to review question content and scoring procedures in order to establish comparability across items before data pooling. We then summarize our findings on how heterogeneity in items both within and across studies might lead to difficulty in interpretations of statistical models. Finally, we offer guidelines for how future research needs to acknowledge and address similar issues in pooling behavioral instruments.

## Methods

### Studies

We identified measures of dementia behavioral symptoms that are used in clinical practice, a national survey, and randomized trials of dementia care interventions. Because our ultimate goal is to develop a statistical crosswalk between common measures of behavioral symptoms, we only needed a single record per participant in studies with longitudinal data. National Institute on Aging funded Alzheimer’s Disease Research Centers submit longitudinal clinical evaluations to the National Alzheimer’s Coordinating Center (NACC) uniform dataset, which includes an assessment of behavioral symptoms. For NACC, we used data from clinical evaluations submitted between September 2005 and May 2020 (*N* = 14,654), and we selected a single random visit for each participant with a dementia diagnosis [1, 4]. The Aging, Demographics and Memory Study (ADAMS) is a US representative survey of cognitive health, in which participants were administered a clinical assessment that includes measures of behaviors [32]. We restricted our analysis to ADAMS Wave A participants with a dementia diagnosis (*N* = 308). Care of Older Persons in Their Environment (COPE) (*N* = 237), the Tailored Activity Program (TAP) (*N* = 60), the Alzheimer’s Quality of Life Study (ALZQOL) (*N* = 88), the Advancing Caregiver Training (ACT) project (*N* = 272), the Resources for Enhancing Alzheimer’s Caregiver Health project (REACH) (*N* = 670), the Adult Day Services Plus study (ADS PLUS) (*N* = 194) are National Institute on Aging funded trials of non-drug dementia care interventions that included measures of dementia related behaviors and are trials in which the study principle investigator was willing to share data. We used baseline data from these trials so that responses would not be confounded by participation in the trial.

Specifically, COPE was a randomized trial to test the effectiveness of a nonpharmacologic intervention that aims to ameliorate physical functioning, quality of life, and behavioral outcomes for people living with dementia [15]. The TAP trial tested a home-based occupational therapy intervention that aimed to reduce behavioral symptoms of people living with dementia [16]. ALZQOL was a randomized trial to assess potentially modifiable risk factors associated with quality of life for persons with dementia and caregivers [12]. ACT was a randomized trial testing a home-based multicomponent intervention targeting environmental and behavioral factors contributing to quality of life of persons with functional disabilities [13, 14]. REACH II was a randomized controlled trial of the effects of a multicomponent intervention on quality of caregiving among caregivers of dementia patients [3]. ADS Plus study was a randomized controlled trial of the effect of a management intervention on quality of caregiving among dementia caregivers, service utilization and institution placement of care recipients [13, 14]. We selected only baseline data from COPE, TAP, ALZQOL, ACT, REACH II, ADS Plus to be merged with other studies.

### Procedure

We acquired codebooks, data entry and test forms, and procedural instructions from each study. We then identified common behavioral instruments and items used within and across studies. We reviewed each individual item to identify its respective behavioral attribute, skip patterns (e.g. questions that are conditional on other items), question stems, response options or scoring types, and theoretical score ranges. This step revealed multiple sources of variation across studies.

Upon reviewing available documentation, we created a crosswalk document that links common items from assorted instruments adopted within and across studies that assess behaviors. As implemented here, a crosswalk is a table that maps common elements between different studies. Each row represents an item of interest (e.g., whether a respondent exhibits false beliefs). The relevant individual test item for a study associated with a construct is placed on the corresponding row in the crosswalk. Items judged by experts to be similar across studies were placed on a row together. Additionally, this crosswalk contained relevant information about each item in each study including the name of source dataset, specific section of the survey, name and version of the instrument being used, study-specific name for each item, question stem, and responses options which included the possible score range. The crosswalk was updated throughout the pre-statistical harmonization procedures listed below. For the purpose of data sharing, this crosswalk will be made available upon request to the senior author.

### Workflow

Establishing a workflow is a process that encompasses all aspects involved in data management. We followed procedures described in *The Workflow of Data Analysis Using Stata,* by J. Scott Long [33]. In our data analysis project, we used a generalizable file structure sharable via a secured online server that can be accessed by team members from different terminals. This structure facilitates reproducible research.

There are nine common folders: 1) Hold then delete, 2) To file, 3) Admin, 4) Documentation, 5) Mailbox, 6) Posted, 7) Private, 8) Readings, and 9) Work. Hold then delete and To file are folders that temporarily hold files so that we can determine the purpose of these files later, as needed. Admin is a folder for budgeting, correspondence with other investigators, IRB paperwork, and the proposal. Posted is probably the most important folder: it contains sub-folders for analysis, data (both source data and data derived with our analytic code; distinguishing between these is especially crucial for purposes of reproducible research), descriptive statistics, figures and interim analyses. Other folders are self-explanatory by their names. Under the Posted folder, there is sub-folder containing the common set of analytic files. Analytic files contain sections of code pertinent to a specific task during the data cleaning and processing. Descriptions of each analytic file are below:

Data management files:

1. Master.do: sets up working directory and calls all files;
2. Preambling.do: sets local macro and global macro to store study-specific item names;
3. Start-latex.do: produces pdfs for reports;
4. Call-source.do: calls data from source data files and processes the raw data minimally, such as reshaping data into long format and generating record ID;
5. Renamevars.do: generates the rename, recode and labeling commands for each item and store them as global macros;
6. Mergestudies.do: calls on renaming, recoding and labeling global macros and merges studies together;
7. Create-variables.do: performs data cleaning so that items have the same values across datasets;
8. Select-cases-for-analysis.do: Identifies data-specific global macros of items for each attribute;

Data analysis files:

9. Model-fitting.do: This program runs IRT analyses of behavioral items in each study separately.
10. Models.do: This program conducts statistical co-calibration via IRT of behavioral items.
11. Sensitivity-analysis.do: This is an optional syntax file for any sensitivity analyses necessary to probe robustness of results.

### Conditional dependency

Responses to some items are conditional on others. For example, answering “yes” to a question about a behavior may prompt, in some datasets, additional questions about frequency or recency of the behavior. These items are inappropriate to be include in statistical harmonization because the items are conditionally dependent on each other. To address this in our datasets, we underwent rigorous efforts to manually review each instrument and items therein. We found that, in this project, items assessing severity or frequency of a behavior were usually conditional on a binary yes/no question assessing presence of a behavior.

### Standardization of item coding

A critical stage of pre-statistical harmonization is to ensure common items, and items that can be made comparable via transformation, are comparable across instruments or studies. One way to achieve this is to code response options in the same way across multiple instruments and studies. For example, ADAMS adopted the Neuropsychiatric Inventory (NPI) as an instrument to measure behavioral outcomes. One specific test item measures whether delusion of danger is present in the participant’s behavior. The question stem is “Does (NAME) believe that (HE/SHE) is in danger--that others are planning to hurt (HIM/HER)?”. Response options include: yes (coded 1), no (coded 5), invalid (coded 6), skipped or not asked because screening symptom not confirmed (coded 96), not assessed/not asked (coded 97), don’t know (coded 98), not applicable/not assessed for this item (coded 99). To standardize response options, we edited the crosswalk column and replaced text descriptions of value labels with stata-readable code that revised values so that a resulting variable would be ready for analysis. For the above example, we set values of 6, 96, 97, 98 and 99 to missing, and values of 5 to 0, such that the final item for this question to be used for analysis has values of 0 (not endorsed) and 1 (endorsed).

### Comparability of items

In addition to manually reviewing each test item, we leveraged several automated approaches to uncover potential cross-study differences in items. We summarized and visualized data by displaying item values specific to each study. For example, we cross-tabulated items and studies conditioning on that item having different minimum and maximum values across studies. Resulting tables can identify items that have different scoring procedures across studies. Another approach to uncover potential sources of heterogeneity is to estimate correlation matrices. These matrices help identify items which have sizable negative intercorrelations with other items, and thus which may need to be reverse coded to be in the same direction as other items. Within each study, we tabulated the frequency of each item to identify skewness and potential outliers. We cycled through every item within each study, filtering out items which have the same minimum value and maximum value within a specific study (indicating no variability). We filtered out items which have maximum value between 90 to 100, or that had negative values because for our scales, such values indicated missing data codes that should be removed prior to analysis via recoding in our crosswalk. In our preliminary IRT analysis, we leveraged both univariate and bivariate residual analysis to identify items that have mismatched model estimated correlation and empirical correlations. Details on how each approach is adopted for a specific harmonization task are given in following sections.

### Missingness and skewness

On top of missingness in responses already coded in original documentation (e.g. In ADAMS, 6 = Invalid, 96 = Skipped, or not asked because screening symptom not confirmed, 97 = Not assessed/Not asked (NPI not completed), 98. = DK (Don’t Know), 99 = Not applicable/not assessed for this item), we paid close attention to other sources of outlying values or skewness. For example, some items represent severe or extremely rare behavioral symptoms (i.e. inappropriate sexual contact), such that the frequency of its being endorsed within a particular study is low. Another possible scenario is when an item is only assessed for a subset of participants, as an artifact of conditional dependency (i.e. an item can only be answered given another item’s response). Sometimes there is little to no variability in responses due to small sample sizes. ALZQOL and TAP are both small randomized trials.

Items which have excessive missingness – which is usually explainable – and skewness in responses could create issues when we run statistical models such as IRT analysis later, mainly because they would have poor correlations with other items.

### Directionality

Items should be consistent in their polarity or direction to facilitate interpretability of harmonized factor analysis. We decided to code “higher” values as indicative of adverse behaviors. If the presence of a certain behavior is considered “worse”, we gave this response option a higher value. The same procedure was undertaken for items that indicate severity or frequency, such that higher values indicate more severe presentations of a behavioral symptom. This step required careful review of question content and response options.

On top of these qualitative review procedures, we adopted an automated approach to identify items in need of reverse coding. Within each study, we ran correlation matrices and flagged potentially problematic negative correlations (*r* < − 0.2). If not by chance, a negative correlation tends to indicate an item may be in need of reverse scoring relative to other items within the same study.

### Scoring type and scales

Other than ensuring consistent directionality, all studies must have the same response levels for a given item (e.g., 4-point Likert scale, 5-point Likert scale, binary no/yes). Discrepant response levels are present when different instruments were used but judged to have common items. We undertook rigorous efforts to parse out presumably comparable items that were subject to differential scoring across studies.

### Model fitting

To check whether that above procedures helped and were not detrimental for statistical co-calibration, in each of the eight studies we estimated two-parameter logistic Item Response Theory (IRT) models. This procedure is akin to testing configural measurement invariance (e.g., [31]). The two-parameter IRT model predicts the probability of an individual endorsing a behavioral symptom or item, as a function of the discrepancy between one’s unobserved level on the underlying trait and the item difficulty parameter, modified by the item discrimination parameter. Item difficulty, akin to a threshold in factor analysis, is the level of the underlying trait at which a randomly selected individual from the population has a 50% probability of endorsing the item. Item discrimination, analogous to a factor loading in factor analysis, describes the strength of association between the item and the underlying trait, or how well the item separates individuals having low and high level of the underlying trait [34, 41]. We scrutinized Mplus output for high residuals between empirical and model-estimated covariances (specifically, standardized residuals greater than 0.3), which would suggest a mismatch between model estimated and empirical correlations [37]. High residuals could imply items measure a similar behavior, or a heretofore undetected problem with conditional independence. Estimation of separate models within each individual study helps establish configural invariance by detecting couplet items that may be subjected to multidimensionality [30]. This procedure helps us to examine whether participants from different studies interpret the behavioral measurement items in a conceptually equivalent way [5]. We use three fit statistics to examine our model fit: root mean square error of approximation (RMSEA), comparative fit index (CFI) and standardized root mean square residual (SRMR). By convention, we adopted a cut-off value of RMSEA less than 0.08 to indicate excellent fit, an RMSEA between 0.05 to 0.08 to indicate mediocre fit. As for CFI, a value greater or equal to 0.90 is indicative of goof fit. A SRMR value lower than 0.08 indicates good fit [28, 29, 35]. Fitting these data to such a model is not conclusive of a problem with harmonization because misfit can also be due to model misspecification, however, we used model fitting as an exploratory approach.

## Results

### Characteristics of study samples

Among the eight samples, NACC has the largest sample size (*n* = 14,564). Thus, the baseline characteristics of our pooled sample are largely driven by participants from NACC. TAP (*n* = 60) and ALZQOL (*n* = 88) are both community-based trials and have the smallest sample sizes. Most study cohorts are balanced in terms of sex of the participants, except for ADAMS and COPE which had predominantly female participants. Mean ages are reasonably comparable across studies. ADAMS has the oldest cohort (85.2 years) and ADS Plus has the youngest (67.3 years). Study cohorts are predominantly White, non-Hispanic origin. Detailed baseline characteristics of study participants of each cohort are available in Table 1.

**Table 1:** Characteristics of study participants

Characteristics	Adult Day Services Plus study (ADS PLUS)	Advancing Caregiver Training (ACT)	Aging, Demographics and Memory Study(ADAMS)	Alzheimer’s Quality of Life Study (ALZQOL)	Care of Older Persons in Their Environment (COPE)	National Alzheimer’s Coordinating Center(NACC)	Resources for Enhancing Alzheimer’s Caregiver Health(REACH II)	Tailored Activity Program (TAP)
**Sample Size**	194	272	308	88	237	14,564	670	60
**Age(in years), mean(SD)**	^a^67.3(12.8)	82.4(8.44)	85.2(6.91)	81.7(8.02)	82.7(8.66)	74.6(9.82)	^a^79.0(9.2)	79.4(9.40)
**Female, n(%)**	99(51.0)	147(54.0)	213(69.2)	46(52.3)	165(69.2)	8064(55.4)	^a^390 (58.2)	26(43.3)
**Race, n(%)**								
**White**	150(77.3)	193(71.0)	224(72.7)	67(76.1)	170(71.7)	11967(82.2)	321(47.9)	46(76.7)
**African American**	29(15.0)	73(26.8)	70(22.7)	20(22.7)	62(26.2)	1716(11.8)	214(31.9)	13(21.7)
**Others**	15(7.73)	6(2.21)	14(4.55)	1(1.14)	5(2.11)	881(6.05)	195(29.1)	1(1.67)
**Ethnicity, n(%)**								
**Hispanic/Latino**	18(9.28)	5(1.84)	23(7.47)	2(2.27)	4(1.69)	1271(8.73)	207(30.9)	2(3.33)
**Non-Hispanic/Latino**	174(89.7)	267(98.2)	285(92.5)	86(97.7)	233(98.3)	13243(90.9)	436(65.1)	58(96.7)
**Unknown**	2(1.03)	0	0	0	0	50(0.34)	27(4.03)	0
**Education, n(%)**								
**Less than high school/GRE**	21(10.8)	^a^25(9.19)	179(58.1)	^a^2(2.27)	^a^7(2.95)	1709(11.7)	314(46.9)	12(20.0)
**High school graduate**	55(28.4)	^a^69(25.4)	70(22.7)	^a^21(23.9)	^a^66(27.9)	3335(22.9)	149(22.2)	19(31.7)
**Some college**	50(25.8)	^a^83(30.5)	32(10.4)	^a^33(37.5)	^a^74(31.2)	2420(16.6)	56(8.36)	4(6.67)
**College degree**	39(20.1)	^a^62(22.8)	14(4.55)	^a^10(11.4)	^a^46(19.4)	3147(21.6)	64(9.55)	14(23.3)
**Post-graduate study**	27(13.9)	^a^33(12.1)	13(4.22)	^a^22(25.0)	^a^44(18.6)	3953(27.1)	37(5.52)	8(13.3)
**Unknown**	2(1.03)	^a^0	0	^a^0	^a^0	0	50(7.46)	3(5.00)

^a^Note: Demographic information of patient with ADRD is not avaliable because it is a study about care giver not care recipient

We identified 59 items from 11 instruments that measure a theoretically similar construct of behavioral symptomatology. Among these items, 29 items are unique to only one study; 4 items are common across 2 studies; 8 items are common across 3 studies; 6 items are common across 4 studies; 4 items are common across 5 studies; 1 item is common across 6 studies; 4 items are common across 7 studies; 3 items are common across 8 studies.

### Conditional dependency

A threat to statistical harmonization is conditional dependency among items. For example, the Neuropsychiatric Inventory-Clinician rating scale (NPI-C) first determines if a behavior is present (e.g. verbal aggression). If so, conditional questions regarding different types of verbal aggression would be asked. During our qualitative review of instruments and item descriptions, we identified 13 items that are conditional upon other items in NACC; 136 conditional items in ADAMS; 38 conditional items in COPE; 42 conditional items in TAP; 120 conditional items in ALZQOL; 39 conditional items in ACT; 57 conditional items in REACH; 34 items in ADSPlus. We excluded these conditional items because they are redundant with other items in a given dataset and cannot depend on other items. As with most statistical methods, dependency among items can inappropriately boost reliability and give a false sense of measurement precision in a psychometric model.

### Missingness and skewness

In reviewing every item within each study, we found that one item, indicating refusal to cooperate with appropriate help or resisting care with daily activities, had no variability in ALZQOL. We thus removed this item from ALZQOL.

### Directionality

Per our qualitative review of question stems and response options, together with automated statistical analysis on correlation matrices, we reverse-coded items within each study to ensure consistent directionality. Specifically, we reverse-coded 22 items in ADAMS; 2 items in COPE; 4 items in TAP; 24 items in ALZQOL; 2 items in ACT; 4 items in REACH.

One example of items that were flagged during qualitative review is one item for poor concentration in the Dementia Quality of Life Instrument (DEMQOL) from ALZQOL. The question stem is: *In the last week, how worried have you been about poor concentration?* The original response options are: *1 = A lot, 2 = Quite a bit, 3 = A little, 4 = Not at all, − 5 = Can’t answer.* Since we decided that for all items, higher values should be indicative of worse or more severe symptomatology, we recoded this item as *3 = A lot, 2 = Quite a bit, 1 = A little, 0 = Not at all,* and *Can’t answer* was recoded as missing. Another example is one item from ADAMS using the Neuropsychiatric Inventory (NPI) assessing whether the participant has enough energy. The question stem is: *Does (HE/SHE) have enough energy?* The original response options are: *1 = Yes, 5 = No, 6 = Invalid, 96 = Skipped, or not asked because screening symptom not confirmed, 97 = Not assessed/Not asked (NPI not completed), 98. = DK (Don’t Know), 99 = Not applicable/not assessed for this item.* Since not having enough energy represents graver symptom and thus should be given a higher score, we recoded the response to *0 = Yes, 1 = No.*

### Scoring types and scales

By reviewing each instrument, we found discrepancies in scoring procedures. For example, the Neuropsychiatric Inventory (NPI), Neuropsychiatric Inventory -Questionnaire (NPI-Q), Neuropsychiatric Inventory-Clinician rating scale (NPI-C), GDS (Geriatric Depression Screening Scale), CDDS (Cornell Depression in Dementia Scale), Care-recipient Behavioral Occurrence and Care-giver Upset (BOUP), Care-recipient Behavioral Occurrence and Care-giver Upset & Problem Behavioral Checklist (BOCGU) used binary coding; the Blessed Dementia Rating (Blessed) instrument adopted categories for scoring a behavioral symptom. Moreover, some instruments such as the Revised Memory and Behavior Checklist, Dementia Quality of Life Instrument (DEMQOL), Dementia Quality of Life Instrument-Proxy (DEMQOL-Proxy) use both binary and categorical scoring. Some items are counts because they were summary scores for behavioral symptoms. We identified four such summary score items and excluded them from our datasets.

One example of a discrepancy in scoring procedures among instruments is: one item from the Blessed Dementia Rating (Blessed) used score 0, 1, 2, 3 to code the presence of no serious behavioral language problem, shouting, cursing, or verbal aggression, with higher scores indicating greater severity; the Care-recipient Behavioral Occurrence and Care-giver Upset (BOUP) instrument used binary codes of 0 and 1 to indicate the presence of such behavior. By cycling through each item and comparing their minimum and maximum values across studies, we identified 20 items that had different scoring or coding procedures across studies. Therefore, we hard-coded some modifications to ensure cross-study consistency in scoring. To be more specific, we kept the 0 option and truncated scores equal or larger than 1, to 1. For studies that coded the lowest score value as 1 and not 0, we shifted all scores down by 1 point.

To reduce the risk of small cells and outliers in analysis, we also performed such truncation for items with small counts in certain cells, if the items were only present in one study. We collapsed one ordinal item into a binary response in REACH II; 3 items in ADAMS; 2 items in COPE; 2 items in TAP.

### Model fitting

We examined model fit statistics, including empirical and model-estimated frequencies and standardized residuals. We leveraged this output to flag items that had a high impact on model fit and items responsible for the high standardized residuals (i.e. greater than 0.3 or smaller than − 0.3). For bivariate residual correlations, we flagged items with residuals greater than 3 or smaller than − 3. Specifically, we identified 4 sets of couplet items in ACT; 6 sets of couplet items in ADAMS; 36 sets of couplet items in ALZQOL; 2 sets of couplet items in COPE; 6 sets of couplet items in REACH II; 13 sets of couplet items in TAP; and 40 sets of couplet items in NACC. Inspection of these items revealed items that are conceptually similar, and are available in Supplemental materials. Such high residuals are potential artifacts of violations of unidimensionality of the set of underlying items. Table 2 shows final model fit statistics for each study’s IRT model. Judging by these criteria, our model fits range from acceptable to excellent in each study.

**Table 2:** Fit statistics of two-parameter IRT models within each study

Study	No. items	RMSEA	CFI	SRMR	Bifactor
**ADSPlus**	12	0.059	0.927	0.1	No
**ACT**	20	0.043	0.868	0.111	Yes
**ADAMS**	18	0.048	0.913	0.11	Yes
**ALZQOL**	25	0.033	0.952	0.123	Yes
**COPE**	19	0.037	0.925	0.103	Yes
**NACC**	23	0.069	0.753	0.123	Yes
**REACH II**	17	0.056	0.85	0.103	Yes
**TAP**	16	0	1	0.118	Yes

## Discussion

Our study’s goal was to describe and document the detailed procedures undertook in pre-statistical harmonization of behavioral instruments, to document our findings uncovered by the procedures in a reproducible manner [7]. To establish comparability of items across instruments and studies, we conducted extensive manual review of instruments and scrutinized raw data using automated procedures. We addressed several sources of heterogeneity across studies. Even when seemingly comparable items were asked across instruments and studies, differences in scoring schemes rendered them essentially non-compatible with each other. Table 3 contains a summary of procedures we undertook and may serve as a checklist for future studies requiring pre-statistical harmonization.

**Table 3:** Recommended procedures

Recommended procedures: • Merge raw data from multiple sources with minimal pre-processing; • Check whether item responses are comparable across sources; • Clean data to establish item comparability: ○ Ensure constant directionality/polarity: ■ Review content and response options; ■ Run correlation matrices, flag items with sizable negative correlations; ■ Reverse code as necessary. ○ Ensure consistency in scoring type and scales: ■ Review response options; ■ Cross-tabulate items across datasets to evaluate whether items have different minimum and maximum values by dataset; ■ Exclude summary scores and counts in favor of more granular data; ■ Truncate, collapse response categories as necessary. ○ Eliminate conditional dependency: ■ Review content and logic flows; ■ Perform parametric modeling, scrutinize output for residuals; ■ Exclude conditional items. ○ Address missingness/skewness: ■Tabulate frequency of each item being endorsed; ■ Filter out items with coded missingness; ■ Filter out items with same min and max within a dataset; ■ Truncate, collapse response categories as necessary; ■Exclude items with no variability. • Establish configural invariance: ○ Estimate parametric models within each dataset; ○ Scrutinize output for residuals; ○ Include residual covariances for items having high covariance residuals.	

We summarize our recommendations for procedures to be taken and potential solutions for issues uncovered in Table 3. We recommend the following three general guidelines to avoid potential pitfalls and streamline the process for applied researchers who intend to develop a data harmonization project. First, establishing comparability of individual items is warranted even when standardized tests and instruments are used because administration differences can result in key cross-study differences. Researchers need to carefully document and scrutinize sources of variance, such as discrepancies in scoring and coding procedures that may lead to erroneous results. To facilitate this review process, obtaining abundance information from the source studies is especially important. Second, use a harmonized coding scheme that is both easy to use and retain all meaningful information [10]. In our study, we used the lowest common denominator (i.e. presence) to select variables that represent comparable behavioral symptoms. This means we discarded additional information regarding other aspects of the symptoms (i.e. frequency or severity). Finally, researchers should be cautious about sources of misfit in statistical models. Surely, misfit is usually attributable to a misspecified model, however in an integrative analysis across multiple data sources such misfit in parametric modeling can also be used to point towards non-equivalent items. Not detecting and accounting for issues around item non-comparability and conditional dependency may lead to failures in achieving acceptable model fit. Our study offers a template for conducting pre-statistical harmonization and fostering reproducibility.

Acknowledging and addressing limitations in raw data are critical steps before conducting data pooling. Our findings have three implications for harmonization of similar datasets involving survey data. First, pooling non-comparable items such as items with reverse polarity and items with different coding schemes across studies may introduce bias by artificially creating variance between individuals from different studies. Second, conditional items or those with too much missingness or skewness may lead to spurious correlations and large residuals in statistical models. Particularly, items identified as being logically conditional on other items are especially problematic because they are highly correlated with each other. On top of that, conditional items usually were assessing the frequency or severity of a present behavior symptom. This is problematic because such count items often have high skewness, especially if a condition is rare. Finally, conditional items provide essentially duplicate information to other items.

This study highlights the importance of each aspect involved in the pre-statistical harmonization process of behavioral instruments. Specifically, conducting careful review of each instrument and item is critical in discovering potential sources of limitations in raw data. This step identified common items to be pooled both across and within studies, uncovered discrepancies in coding schemes, detected skip patterns. Performing preliminary IRT analysis within each study helped ensure reasonable model fit before pooling across studies. This step detected items that may violate the local independence or the unidimensionality assumption of IRT models [27]. Regardless of the statistical methods of harmonizing behavioral instruments of choice, detailed approaches described in this paper to uncover and tackle issues that are specific to harmonizing behavioral instruments are important to consider before carrying out the analysis.

After conducting pre-statistical harmonization, one conducts statistical harmonization to derive scores for a construct that are commonly scaled across multiple data sources. This score or set of scores can then be used for substantive research questions [17, 19–25, 36].

One limitation of the current study is that we did not combine items that indicate frequency or severity of a behavioral symptom with the screener item (i.e. item that indicate presence of the behavioral symptom). Instead, we excluded all conditionally dependent items. Additionally, we distilled all ordinal items into binary scale. Using indicator coding simplifies our analysis, but may lead to loss of resolutions and item quality. Another potential limitation is that identifying common items across datasets can be subjective. However, we leveraged expert reviews of items to assign items, which is considered state-of-the-art. In our next stage of analysis, after deriving a factor score for each participant, we were able to quantify the amount of error based on the quality and missingness of the respective item in a given study battery. We found a considerable number of participants have imprecisely estimated factor scores, especially in ADSPlus, ADAMS and REACH II. This observation could be a reflection of the inherent nature of psychometric measurements used to assess problematic dementia behaviors. However, this warrants careful interpretation of the harmonized factor scores, and may point to the need of sensitivity analysis in the future.

## Conclusions

Data harmonization is an essential step towards effective use of existing data. In this study of pre-statistical harmonization, we pooled data on measures and items of dementia related behavioral symptoms captured in clinical assessments, a national survey, and randomized trials of non-drug dementia care interventions. An important next step is to reproduce the pre-statistical harmonization procedures described in this paper in other domains of interest, such as measures on functional and cognitive abilities of dementia patients across datasets.

## Supplementary Information

**Additional file 1.**

## Data Availability

The datasets used during the current study are available in JHU OneDrive. The crosswalk we created, which can be used to reproduce the pre-statistical harmonization described in this manuscript, can be obtained from the corresponding author upon request.

## Abbreviations

ACT: The Advancing Caregiver Training project
ADAMS: The Aging, Demographics and Memory Study
ADS Plus: The Adult Day Services Plus study
ALZQOL: The Alzheimer’s Quality of Life study
Blessed: The Blessed Dementia Rating instrument
BOUP: The Care-recipient Behavioral Occurrence and Care-giver Upset instrument
BOCGU: The Care-recipient Behavioral Occurrence and Care-giver Upset & Problem Behavioral Checklist
CDDS: The Cornell Depression in Dementia Scale
COPE: The Care of Older Persons in Their Environment study
DEMQOL: The Dementia Quality of Life Instrument
DEMQOL-Proxy: The Dementia Quality of Life Instrument-Proxy
GDS: The Geriatric Depression Screening Scale
IRT: Item Response Theory
NACC: The National Alzheimer’s Coordinating Center
NPI: The Neuropsychiatric Inventory
NPI-C: The Neuropsychiatric Inventory-Clinician rating scale
NPI-Q: The Neuropsychiatric Inventory-Questionnaire
REACH: The Resources for Enhancing Alzheimer’s Caregiver Health project
TAP: The Tailored Activity Program
UDS: The Uniform Data Set
